# The Efficacy of Pain Neuroscience Education on Active Rehabilitation Following Arthroscopic Rotator Cuff Repair: A CONSORT-Compliant Prospective Randomized Single-Blind Controlled Trial

**DOI:** 10.3390/brainsci12060764

**Published:** 2022-06-10

**Authors:** Hyunjoong Kim, Seungwon Lee

**Affiliations:** 1 Department of Physical Therapy, Graduate School of Sahmyook University, 815, Hwarang-ro, Nowon-gu, Seoul 01795, Korea; doong18324@gmail.com; 2 Department of Physical Therapy, College of Health and Welfare, Sahmyook University, 815, Hwarang-ro, Nowon-gu, Seoul 01795, Korea

**Keywords:** pain neuroscience education, rotator cuff repair, postoperative rehabilitation, physical therapy

## Abstract

Pain neuroscience education (PNE), a modern educational therapy, has been reported to be effective in pain control by reducing fear of movement. This study investigated the effects of additional PNE on a physical therapy rehabilitation protocol (PTRP) following arthroscopic rotator cuff repair (ARCR). In this single-blind, randomized controlled trial, 34 patients who had undergone ARCR were randomly allocated (1:1) into two groups: PNE (PTRP plus PNE) and PTRP. PTRP was performed five times a week, for four weeks, 115 min per session (physical agents, manual therapy, and exercises), and PNE was performed twice at the beginning (face-to-face PNE) and end (non-face-to-face) of the PTRP. The outcome measures were measured four times for pain intensity, pain cognition, and shoulder function; two times for a range of motion; and once for satisfaction. No significant difference in pain intensity was observed between the groups. However, in pain cognition, the Tampa Scale for Kinesiophobia avoidance showed a significant interaction between time and group, and PNE showed a higher effect size than PTRP in the post-test and follow-up in several variables. In conclusion, the significant improvement in avoidance in postoperative rehabilitation suggests that there is a partially positive benefit in terms of pain, range of motion, and shoulder function in ARCR patients.

## 1. Introduction

Disorders of the rotator cuff and muscles around the shoulder, which are considered the most common causes of shoulder pain, are also the most common causes of musculoskeletal pain. In addition, rotator cuff disorders, including rotator cuff tears, are considered degenerative diseases, and their incidence increases with age [1]. Primary care for patients with shoulder pain and rotator cuff tear mainly involves changes in usual activities, use of analgesics, corticosteroid injections, and physical therapy [2,3]. If conservative treatment fails, surgical treatment may be considered [4,5].

After arthroscopic rotator cuff repair (ARCR), various rehabilitation protocols have been proposed; in principle, the postoperative rehabilitation phase proceeds according to the tendon healing process. Based on recent research trends, early and delayed rehabilitation are considered controversial [6], and approaches for them are still limited [7,8]. The existing general rehabilitation protocol minimizes active motion for 4 weeks after surgery and focuses on conscious muscle control of the upper extremities and trunk [9,10].

However, in clinical settings, patients cannot easily control their pain. It has been reported that pain neuroscience education (PNE), a modern educational therapy method, is effective in pain control by reducing fear of movement based on an understanding of neurophysiology [11]. Studies on PNE are mainly based on the beliefs and cognitions of patients about chronic pain that affect the patient’s pain experience and treatment outcomes [12,13]. However, recent research trends have changed to identify the potential benefits in non-chronic pain conditions.

We hypothesized that education could potentially reduce the likelihood of developing chronic pain and disability for patients with acute, subacute, preoperative, and prior pain experiences (healthy individuals). In a related study conducted in the United States, patients who received preoperative PNE before their back surgery and total knee arthroplasty showed significant improvement in health at six months, one year, and three years of follow-up compared to those who did not receive preoperative PNE [14,15,16].

This study aimed to investigate the effect of additional PNE on pain intensity, pain cognition, range of motion, shoulder function, and treatment satisfaction in the rehabilitation protocol after ARCR.

## 2. Materials and Methods

### 2.1. Study Design

**Figure 1:**
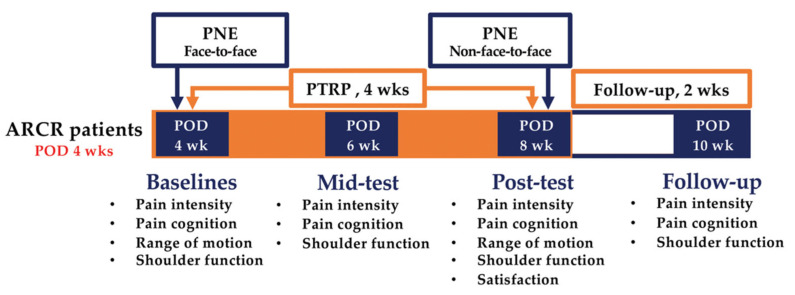
Schematic of the experimental design. ARCR: arthroscopic rotator cuff repair; PNE: pain neuroscience education; POD: postoperative day; PTRP: physical therapy rehabilitation protocol.

This study was a two-arm, parallel, single-blind randomized controlled trial with a longitudinal prospective design. The study was conducted from February to August 2021, and the protocol was registered in January (ClinicalTrial.gov.: NCT0475311). Figure 1 shows the data collection and research procedures.

### 2.2. Participants and Ethics

This study included patients admitted to The Better Hospital (Gwangju, Republic of Korea) for postoperative rehabilitation after ARCR. Potential participants were recruited autonomously through the sports rehabilitation center bulletin board. Assessment for eligibility was based on some inclusion and exclusion criteria [17,18].

#### 2.2.1. Inclusion Criteria

- Adults aged >18 years;
- Four weeks after ARCR;
- Willing to participate in the study.

#### 2.2.2. Exclusion Criteria

- Older adults (age > 65 years);
- Unable to receive education remotely;
- Additional tendon augmentation in ARCR;
- History of surgery on the same shoulder before ARCR;
- Osteoarthritis findings in the shoulder joint;
- Mental health and cognitive problems to the extent that they cannot understand the guidelines for assessment and/or intervention.

#### 2.2.3. Ethics

Before starting the study, the researcher (H.K.) directly explained the purpose, significance, importance, and procedure of the study to all participants and provided information on the risks and inconveniences that may occur during the experiment and the risk prevention plan. A sufficient explanation was also provided in writing. Subsequently, the participant filled out the informed consent form (ICF). The ICF avoided medical terminology and was written in an easy-to-understand manner for participants. In addition, the confidentiality and anonymity of the participants’ personal information were guaranteed, and the researcher provided answers to the research participants’ questions at all times. The participants were informed that they could withdraw from participating in the study at any time.

All participants were informed of the purpose and procedures of the ethical standards of the Declaration of Helsinki before the study. The institutional review board of Sahmyook University approved this study (2-1040781-A-N-012021010HR).

### 2.3. Sample Size

Sample sizes were calculated using different values from the simple shoulder test (SST) in the study by Mazzocca et al. [19]. Calculations were performed using G*power 3.1 (Franz Faul, University Kiel, Germany). The settings configured in the software were effect size f(v) = 0.23, power = 0.80, number of groups = 2, measure = 4. The required number of participants was calculated to be 28. Thirty-four participants were recruited, taking into account dropouts from the total number of participants.

### 2.4. Randomization and Blinding

The enrolled participants were randomly allocated to two equal-sized blocks for PNE and the physical therapy rehabilitation protocol (PTRP) using a random allocation software (Isfahan University, Isfahan, Iran). Additionally, the identification code was randomly generated using two digits. The single-blind interventions were scheduled differently. The educational group was held in the hospital cafeteria on a separate schedule. However, the assessor (H.K.) was not blinded during the four tests carried out (baseline, mid-test, post-test, and follow-up).

### 2.5. Intervention

The two groups were subjected to PNE (PNE plus PTRP) and PTRP. As shown in Figure 1, PNE was performed twice (face-to-face and non-face-to-face) for 30 min each, and PTRP was performed five times a week for four weeks, 115 min per session (physical agent: 35 min; manual therapy: 30 min; exercises: 50 min).

#### 2.5.1. Pain Neuroscience Education

PNE aims to reconceptualize pain perception from a biomedical or structural model to a biopsychosocial pain model through education on the neurophysiological aspects of pain. Participants assigned to the PNE group underwent a baseline assessment, followed by pain education by a physical therapist (H.K.) in groups of 1–4 patients. The educational content of PNE consisted of the following [15,16,20,21]:

- Definition and types of pain;
- Neurophysiology of pain;
- No reference to anatomical or pathoanatomical models;
- No discussion of emotional or behavioral aspects of pain;
- Nociception and nociceptive pathways;
- Mechanism of pain control;
- Peripheral sensitization;
- Pain alarm system;
- Central sensitization;
- Plasticity of the nervous system;
- Treatment cases in pain neuroscience education;
- Shoulder biomechanics;
- Arthroscopy rotator cuff repair details and procedure.

The program was constructed through a meta-analysis on the effect of PNE on pain and kinesiophobia, which we previously reported [11]. In face-to-face PNE, friends and family were allowed to accompany the patient in the hospital cafeteria to reduce the burden of education and improve fidelity [22]. The PNE was conducted using audiovisual materials centered on easy-to-understand metaphors and images for 30 min and a summarized PNE handout was distributed. Non-face-to-face education was performed by providing individual video links after 4 weeks of PTRP.

#### 2.5.2. Physical Therapy Rehabilitation Protocol

The rehabilitation protocol in Table 1 was designed to evaluate the effectiveness of postoperative rehabilitation in patients [23,24,25]. The rehabilitation protocol was a physical therapy intervention that consisted of physical agents, manual therapy, and exercise. As in our previous study, five sessions a week with 115 min per session (physical agent: 35 min; manual therapy: 30 min; exercises: 50 min) were conducted for four weeks.

### 2.6. Outcomes

Variables were measured four times at two-week intervals from the 4th to the 10th week since postoperative day (POD) (Figure 1). An assessor divided the measured variables largely into evaluations and self-report questionnaires. Mid-test (POD 6 wk) and follow-up (POD 10 wk) were measured using self-report questionnaires.

#### 2.6.1. Pain Intensity

The primary outcome measured in this study was pain intensity. Pain intensity was evaluated by dividing pain into usual and worst pain. The numeric pain rating scale (NPRS) consists of 11 points, ranging from 0 (no pain) to 10 (most severe pain imaginable) [26]. NPRS has a high score in test–retest reliability (intraclass correlation coefficient (ICC) = 0.74) and a minimal clinically important difference (MCID) of 1.1 points [26,27].

#### 2.6.2. Pain Cognition

Pain cognition was measured using the Korean version of the Pain Catastrophizing Scale (K-PCS) and the Tampa Scale for Kinesiophobia (TSK-11). The K-PCS is a questionnaire assessment tool that evaluates catastrophic thoughts and emotions related to pain [28,29]. On a 5-point scale with a range of 13 items, a score of 0 means “never” and a score of 4 means “always”. A high score indicates the severity of pain [30]. The minimal detectable change (MDC) for the PCS is 9.1 [31].

The TSK-11 is a questionnaire assessment tool used to evaluate kinesiophobia, which refers to a fear of movement [32,33]. On a 4-point scale of 11 items, 1 point means “totally agree” and 4 means “completely disagree”. The higher the score, the greater the avoidance response due to the fear of movement [34]. The MDC of TSK-11 is 5.64 [35].

#### 2.6.3. Range of Motion

Shoulder joint range of motion (ROM) was measured according to international guidelines using a goniometer [36]. ROM measurement using a goniometer has excellent intra-inspector reliability (ICC = 0.91–0.99) [37]. Active flexion, scaption flexion, abduction, horizontal adduction, external rotation, and internal rotation were measured [18].

#### 2.6.4. Shoulder Function

Shoulder function was evaluated using the Korean version of the disabilities of the arm, shoulder, and hand (K-DASH), the Korean version of the shoulder pain and disability index (K-SPADI), and SST as self-report questionnaires.

The K-DASH is a widely used tool for evaluating patients with shoulder joint disorders [38]. It consists of 30 items and the score ranges from 0 to 100, with 0 indicating no disability. The reported ICC (2, 1) is 0.96 [39,40] and the MCID is 10.2 points [41].

The K-SPADI measures the level of disability perceived by the patient. It consists of a subscale of five items measuring pain and a subscale of eight items measuring disability. The total score is 100 points; the higher the score, the greater the disability [42,43,44]. The SPADI has a test–retest reliability (ICC) of 0.89 [45], and the reported MCID ranges from 8 to 13.2 [41,42,46].

The SST consists of daily life-related items regarding the shoulder joint. It consists of 12 items with “yes” or “no” responses. A higher score indicates shoulder joint dysfunction in a state in which physical performance is impossible. The inter-rater reliability (r) of the SST is 0.85 [47], and the MCID is 2 points [48].

#### 2.6.5. Treatment Satisfaction

The method introduced by Tashjian et al. [49] was used to measure treatment satisfaction using the visual analog scale (VAS) for patients with rotator cuff repair. Participants marked their experience ranging from “not at all satisfied” to “very satisfied” on a 10 cm line. Satisfaction was evaluated only at the end of the 4-week intervention.

### 2.7. Data Analysis

All statistical analyses were performed using IBM SPSS Statistics version 25.0 (IBM Corp., Armonk, NY, USA). To test homogeneity for the two parallel arms, a chi-square test was performed for categorical variables, and an independent t-test was performed for continuous variables. The general characteristics of the participants were expressed using descriptive statistics, and two-way repeated-measures analysis of variance was performed to determine the differences between groups according to the time of measurement. An independent t-test was performed to determine when the interaction between groups appeared over time. When an interaction was detected, a post hoc test was performed using the Bonferroni test. To analyze the effect size of variables for each group, Cohen’s d was used when only two measurements were performed, and partial eta squared ($\eta_{p}^{2}$) was used when four measurements were performed [50]. All statistical significance levels (α) were 0.05, and in the post hoc test, they were set to 0.0125 according to the number of measurements.

## 3. Results

**Figure 2:**
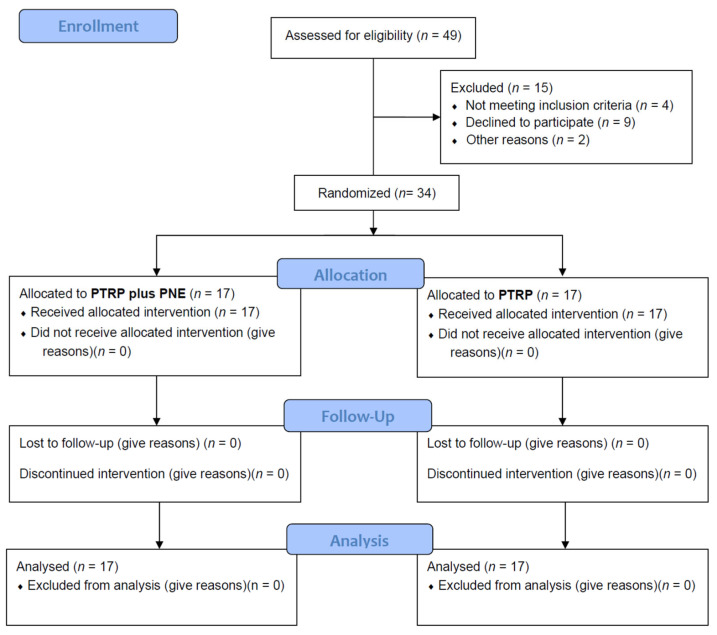
CONSORT flow diagram. CONSORT: Consolidated Standards of Reporting Trials; PNE: pain neuroscience education; PTRP: physical therapy rehabilitation protocol.

Figure 2 shows a flowchart of this study based on the Consolidated Standards of Reporting Trials (CONSORT) guidelines. Forty-nine potential participants were screened, and fifteen participants were excluded. All 34 enrolled participants were analyzed without any dropouts.

### 3.1. General Characteristics of the Participants

Table 2 presents the participants’ general characteristics. There were no significant differences between the groups in terms of sex, affected side, age, height, weight, and body mass index (*p* < 0.05). However, in ARCR, there were significant differences between groups in capsular release and biceps tenodesis (*p* < 0.05).

### 3.2. Pain Intensity

**Figure 3:**
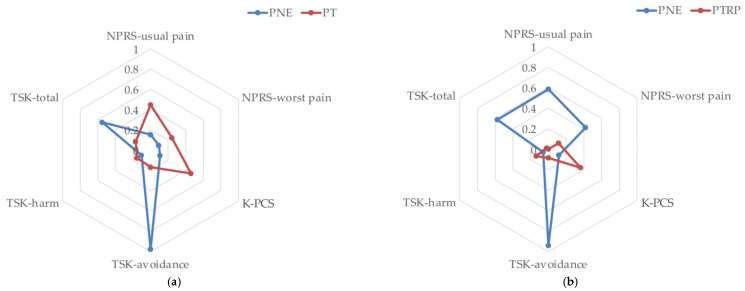
A radar chart of pain intensity and cognition using Cohen’s d effect size: (**a**) post-intervention effect (post-test—baselines); (**b**) carryover effect (follow-up—baselines). NPRS: numeric pain rating scale; K-PCS: Korean version of the pain catastrophizing scale; PNE: pain neuroscience education; PTRP: physical therapy rehabilitation protocol; TSK: Tampa Scale for Kinesiophobia.

There was no interaction between time and group for either usual or worst pain (*p* > 0.05, *F* = 2.455). However, in the case of usual pain, the pain intensity over time showed significant improvement (*p* < 0.05, *F* = 3.182), and the effect size was medium ($\eta_{p}^{2}$ = 0.071) (Table 3) (Figure 3).

### 3.3. Pain Cognition

The results for pain cognition are presented in Table 3 and Figure 3. In the K-PCS results, there was no interaction between time and group (*p* > 0.05, *F* = 1.267), and no statistically significant difference with time was found (*p* > 0.05, *F* = 1.611). The effect size was small ($\eta_{p}^{2}$ = 0.038). The TSK-avoidance results showed a significant interaction between time and group (*p* < 0.01, *F* = 5.650). A statistically significant difference with time was also found (*p* < 0.01, *F* = 4.469), and the effect size was large ($\eta_{p}^{2}$ = 0.150). In the TSK-harm results, there was no interaction between time and group (*p* > 0.05, *F* = 0.097), and no statistically significant difference was found with time (*p* > 0.05, *F* = 0.931). Similarly, in the TSK-total results, there was no interaction between time and group (*p* > 0.05, 2.191), and no statistically significant difference with time was found (*p* > 0.05, *F* = 0.982). However, the effect size was medium ($\eta_{p}^{2}$ = 0.064).

### 3.4. Range of Motion

There was no difference between the groups in all measured shoulder ROM variables (*p* > 0.05). However, in flexion, scapular flexion, abduction, and external rotation, the increase in ROM was larger in the PNE group than in the PTRP group. This increased ROM was not observed in horizontal adduction and internal rotation (Table 4).

### 3.5. Shoulder Function

**Figure 4:**
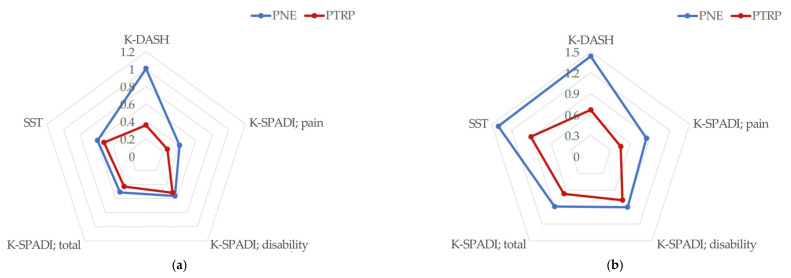
A radar chart of the shoulder function using Cohen’s d effect size: (**a**) post-intervention effect (post-test—baselines); (**b**) carryover effect (follow-up—baselines). K-DASH: Korean version of the disabilities of the arm, shoulder, and hand; PNE: pain neuroscience education; PTRP: physical therapy rehabilitation protocol; K-SPADI: Korean version of the shoulder pain and disability index; SST: simple shoulder test.

There was no interaction between time and group in any of the results for shoulder function (*p* > 0.05). However, a significant improvement in function over time was observed in all results (*p* < 0.001). For the mean difference, the functional improvement of the PNE group was outstanding (Figure 4). For the effect size, there was a medium effect in K-DASH ($\eta_{p}^{2}$ = 0.064) (Table 3).

### 3.6. Treatment Satisfaction

On comparing treatment satisfaction in the post-test, the difference in the average score was 0.47, indicating no statistically significant difference (*p* > 0.05, *t* = −0.922) (Table 4).

## 4. Discussion

PNE, which has previously been studied only in central sensitization in chronic pain, was studied for use in subacute conditions for the first time. We recognized that difficulty in pain control and functional improvement in postoperative rehabilitation is a chronic problem. Therefore, we investigated the beneficial effects of additional PNE on pain intensity, pain cognition, range of motion, shoulder function, and treatment satisfaction during postoperative rehabilitation following ARCR. Our results were generally better than those of a previous study [23,24,25] that investigated the effect of PTRP alone. Additionally, a significant interaction between time and group was found in the avoidance subscale of pain cognition (F = 5.650, $\eta_{p}^{2}$ = 0.150).

The primary outcome measure was pain intensity, and no interaction between time and group was observed with regard to this (*p* > 0.05). There was no significant decrease in PTRP and PTRP plus PNE compared to the reported MCID (1.1 points) in previous studies. However, the effect size was found to be medium only for usual pain ($\eta_{p}^{2}$ = 0.071). These results may be useful for postoperative pain control. In a digitally assisted versus conventional home-based rehabilitation study for rehabilitation following ARCR, pain was maintained or increased for up to 12 weeks [51]. In addition, when comparing our results with those of a study that used steroid injections for pain control, our results were much higher at 6 weeks [52]. These results are interpreted as uncontrolled pain during the tissue healing process because POD 4 week is when the proliferative stage ends and the maturation and remodeling stage begins [53].

In this study, no interaction between time and group was found for any of the variables except TSK-avoidance in pain cognition. However, the PTRP and the additional PNE showed superior results compared to other studies, in pain cognition except for PCS and TSK-harm showed a larger effect size than the PTRP-only group in the results of the individual analysis of each variable (Table 4) (Figure 3 and Figure 4). Similarly, the results of shoulder function were not statistically significant (*p* < 0.05), but additional PNE showed a larger effect size on the improvement of shoulder function. These results were inferred from TSK-avoidance, which showed the only significant difference for measured variables. Although there was no significant difference in pain intensity, additional PNE showed better overall scores for shoulder function and active ROM (shoulder flexion, scaption flexion, abduction, and external rotation). This is because the event of surgery enhances fear of pain, and because pain is caused by tissue damage, a certain amount of time is required for pain control. Therefore, shoulder active motion and function were further improved in patients who received pain education; less avoidance of fear and low awareness of pain-induced disability were reported [54]. In addition, considering that the levels of fear avoidance and pain interference are risk factors for sustaining pain [55], it can be inferred from the results obtained that additional PNE is more beneficial in the follow-up of pain intensity (Figure 4).

This is the first randomized controlled trial to perform PNE for postoperative pain other than central sensitization. When compared to PTRP, no significant difference was found in all variables except TSK-avoidance. However, when the effect sizes were compared, additional PNE showed more positive results for most variables. Therefore, it is worth analyzing the relationship between avoidance and other variables. To relieve the fear of movement in active rehabilitation by starting active motion, our PNE had additional information on ARCR and shoulder biomechanics. This was based on the pain–tension–fear cycle reported in other pain theories [56,57]. In other words, PNE is essential in postoperative rehabilitation to control increased pain due to fear of movement during the immobilization phase after ARCR. This is because physiological arousal owing to autonomic nervous system activation affects pain fear and avoidance behavior, contributing to the maintenance of chronic pain [58]. In addition, in a functional magnetic resonance imaging study of patients with chronic neck pain and healthy adults, the right dorsolateral prefrontal cortex, an emotional factor of pain, the anterior insular cortex, and increased functional connectivity were significantly related to an increase in TSK [59,60]. The insular cortex is responsible for various higher-order cognitive processes and information related to the state of the body during other emotional processes [61]. It has been reported that discomfort caused by tonic pain is encoded in the insular cortex and contributes to persistent pain [62]. This is consistent with the contribution of the insular cortex to the construction of a unique signature/fingerprint of pain experience [63].

Therefore, we assumed that the pain caused by avoidance from fear of movement was a tonic pain due to tension. Based on the association between the insular cortex and TSK in this cycle, this was thought to contribute to the pain experience. Although there was no clear difference between the control group (PTRP) and the PNE group, there was a significant difference in avoidance, a subscale of TSK, and a high effect size was observed in other variables (excluding horizontal adduction and internal rotation).

Our study has several limitations. First, an in-depth analysis was not conducted because the measurement of central sensitization suitable for pain education and psychological measurement after surgery were not performed. Second, a long-term study is needed because the intervention and follow-up periods in the postoperative rehabilitation study were relatively short. Third, the TSK-11 and SST were directly translated and performed by the researcher. Fourth, it may be difficult for some to understand PNE by listening alone, so a tool is needed to check the level of understanding of the educational content. In future studies, it will be necessary to add educational content suitable for various patient groups and each condition, as well as PNE for postoperative patients.

As a clinical implication of the results of this study, this was the first study in which it was confirmed that PNE for neuroplastic pain control in postoperative pain (acute or subacute pain) control can partially contribute. Postoperative joint immobilization or passive range of motion exercise is required in the maximum protective phase (acute condition) after surgery. Although the period differs depending on the affected tissue, in rehabilitation, an active range of motion exercise that requires voluntary movement after a certain period is more effective for tissue healing. However, the pain experience caused by postoperative pain may further affect the pain–tension–fear cycle, contributing to an increase in pain. Therefore, postoperative PNE could be provided as an additional treatment option for the improvement of chronically progressive pain and decreased function in postoperative patients.

## 5. Conclusions

In conclusion, we performed a randomized controlled trial to compare the effect of additional PNE on ARCR patients with PTRP in active rehabilitation after a certain immobilization phase. Our results showed a decrease in TSK avoidance compared to PTRP after 4 weeks of intervention and in the follow-up evaluation two weeks later. Furthermore, additional PNE showed better effect sizes in pain, range of motion, and shoulder function in ARCR patients. We propose, for the first time, the potential impact of PNE on postoperative rehabilitation.

## Figures and Tables

**Table 1:** Physical therapy rehabilitation protocol.

Types	Component	Dosage
Physical agents	Superficial heat therapy	15 min per session, 5 sessions per week
	Microwave therapy	5 min per session, 5 sessions per week
	TENS	15 min per session, 5 sessions per week
Manual therapy	Soft tissue mobilization	20 min per session, 5 sessions per week
	Joint mobilization	10 min per session, 5 sessions per week
Exercises	ROM exercise	30 min per session, 5 sessions per week
	Therapeutic exercise	20 min per session, 5 sessions per week

CPM, continuous passive motion; POD, postoperative day; ROM, range of motion; TENS, transcutaneous electrical nerve stimulation.

**Table 2:** General characteristics of the participants.

Variables	PNE (n = 17)	PTRP (n = 17)	X^2^/t
	Mean ± SD	Mean ± SD	
General characteristics			
Sex (male, %)	7 (41.18)	6 (35.29)	0.125
Affected side (Left, %)	6 (35.29)	7 (41.18)	0.125
Age (years)	51.12 *±* 5.64	51.82 *±* 4.85	−0.391
Height (cm)	163.59 *±* 7.53	162.18 *±* 6.07	0.601
Weight (kg)	63.88 *±* 8.87	64.82 *±* 9.38	−0.301
BMI (kg/m^2^)	23.79 *±* 2.10	24.55 *±* 2.42	−0.971
Arthroscopic rotator cuff repair			
Supraspinatus (n)	7	9	0.472
Subscapularis (n)	12	10	0.515
Capsular release (n)	7	13	4.371 *
Biceps tenodesis (n)	16	11	4.497 *
SAD (n)	17	17	-

BMI, body mass index; PNE, pain neuroscience education; PTRP, physical therapy rehabilitation protocol; SAD, subacromial decompression; SD, standard deviation. * *p* < 0.05, statistically significant difference.

**Table 3:** Differences between groups by measurement time point for pain intensity, pain cognition, and shoulder function.

Variables		Baselines	Mid-Test	Post-Test	Follow-Up	Time ^(a)^ F (*p*)	Time × Group ^(a)^	
		Mean ± SD	Mean ± SD	Mean ± SD	Mean ± SD		F (*p*)	Effect Size ^(b)^
Pain intensity								
Usual pain	PNE	4.12 ± 1.76	3.41 ± 2.00	3.82 ± 2.07	3.06 ± 1.85	3.182 (0.027)	2.455 (0.068)	0.071
	PTRP	3.41 ± 1.46	3.88 ± 1.80	4.12 ± 1.69	3.41 ± 1.80			
Worst pain	PNE	5.65 ± 2.64	4.76 ± 2.46	5.41 ± 2.37	4.65 ± 2.09	2.399 (0.073)	1.672 (0.178)	0.050
	PTRP	4.82 ± 2.24	5.29 ± 1.45	5.35 ± 2.12	4.59 ± 1.77			
Pain cognition								
K-PCS	PNE	19.24 ± 13.37	18.76 ± 13.11	20.71 ± 13.78	17.71 ± 12.28	1.611 (0.211)	1.267 (0.286)	0.038
	PTRP	17.06 ± 11.36	21.06 ± 10.19	22.06 ± 10.35	21.00 ± 10.34			
TSK-avoidance	PNE	13.59 ± 3.24	12.24 ± 2.73	10.41 ± 3.26 **	10.65 ± 3.00 *	4.469 (0.006)	5.650 (0.001)	0.150
	PTRP	13.29 ± 3.53	13.24 ± 3.60	13.82 ± 2.74	13.00 ± 3.34			
TSK-harm	PNE	9.94 ± 2.14	9.71 ± 2.37	10.18 ± 2.40	10.06 ± 1.89	0.931 (0.417)	0.097 (0.942)	0.003
	PTRP	9.94 ± 3.07	9.59 ± 2.81	10.35 ± 2.18	10.35 ± 2.96			
TSK-total	PNE	23.53 ± 5.16	21.94 ± 4.90	20.59 ± 5.53	20.71 ± 4.65	0.982 (0.405)	2.191 (0.121)	0.064
	PTRP	23.24 ± 6.34	22.82 ± 6.06	24.18 ± 4.65	23.35 ± 6.13			
Shoulder function								
K-DASH	PNE	73.92 ± 14.75	59.02 ± 14.40	58.14 ± 16.63	52.35 ± 15.29	22.342 (0.000)	2.190 (0.118)	0.064
	PTRP	71.18 ± 20.36	64.17 ± 16.30	64.41 ± 17.57	58.97 ± 16.00			
K-SPADI -pain	PNE	61.18 ± 26.95	51.06 ± 24.65	50.82 ± 24.21	40.24 ± 22.54	15.245 (0.000)	1.370 (0.257)	0.041
	PTRP	56.94 ± 26.26	53.29 ± 21.77	50.47 ± 23.92	45.41 ± 24.42			
K-SPADI -disability	PNE	54.41 ± 28.11	41.40 ± 22.52	39.71 ± 24.03	31.91 ± 21.31	16.431 (0.000)	0.080 (0.971)	0.002
	PTRP	54.49 ± 27.68	43.97 ± 23.35	40.88 ± 24.89	34.56 ± 23.41			
K-SPADI -total	PNE	57.01 ± 27.41	45.11 ± 22.79	43.98 ± 23.58	35.11 ± 21.42	17.917 (0.000)	0.366 (0.778)	0.011
	PTRP	55.43 ± 26.83	47.56 ± 20.80	44.57 ± 23.58	38.73 ± 23.40			
SST	PNE	8.59 ± 2.92	6.06 ± 1.82	6.71 ± 3.46	4.82 ± 2.43	16.056 (0.000)	1.469 (0.236)	0.044
	PTRP	8.18 ± 1.70	7.12 ± 1.69	6.82 ± 3.34	5.94 ± 3.03			

^(a)^ Repeated-measures analysis of variance, ^(b)^ Partial eta squared. K-DASH, the Korean version of the disabilities of the arm, shoulder, and hand; K-PCS, the Korean version of the pain catastrophizing scale; PNE, pain neuroscience education; PTRP, physical therapy rehabilitation protocol; K-SPADI, the Korean version of the shoulder pain and disability index; SST, simple shoulder test; TSK, Tampa Scale for Kinesiophobia. * *p* < 0.05, ** *p* < 0.01, statistically significant difference.

**Table 4:** Comparison between groups for a range of motion and treatment satisfaction.

Variables		Baselines	Post-Test	t (95% CI) ^(a)^	Effect Size ^(b)^
		Mean ± SD	Mean ± SD		
Range of motion					
Flexion	PNE	147.94 ± 29.00	177.94 ± 7.30	−4.604 *** (−43.814–−16.186)	1.419
	PTRP	147.76 ± 32.94	172.06 ± 17.77	−2.953 ** (−41.737–−6.851)	0.918
	t (95% CI) ^(c)^	−0.017 (−21.856–21.503)	−1.262 (−15.566–3.609)		
Scaption flexion	PNE	145.29 ± 30.69	177.65 ± 7.31	−4.715 *** (−46.899–−17.807)	1.451
	PTRP	135.18 ± 34.78	169.41 ± 20.53	−3.800 ** (−53.335–−15.136)	1.199
	t (95% CI)	−0.899 (−33.035–12.800)	−1.558 (−19.262–2.791)		
Abduction	PNE	132.35 ± 35.58	169.41 ± 25.85	−4.039 ** (−56.511–−17.606)	1.192
	PTRP	125.88 ± 40.44	154.71 ± 37.27	−2.397 * (−54.314–−3.333)	0.741
	t (95% CI)	−0.495 (−33.080–20.139)	−1.337 (−37.222–7.811)		
Horizontal adduction	PNE	107.94 ± 25.38	121.18 ± 13.17	−2.228 * (−25.827–−0.643)	0.655
	PTRP	93.82 ± 34.26	117.65 ± 20.47	−2.496 * (−44.056–−3.591)	0.844
	t (95% CI)	−1.365 (−35.179–6.944)	−0.598 (−15.556–8.497)		
External rotation	PNE	64.12 ± 14.50	80.88 ± 9.05	−5.647 *** (−23.058–−10.471)	1.387
	PTRP	66.76 ± 16.10	77.35 ± 12.64	−3.139 ** (−17.738–−3.438)	0.732
	t (95% CI)	0.504 (−8.055–13.349)	−0.936 (−11.210–4.152)		
Internal rotation	PNE	45.29 ± 16.53	53.82 ± 18.50	−2.792 * (−15.005–−2.054)	0.486
	PTRP	41.18 ± 13.52	50.00 ± 16.30	−2.839 * (−15.411–−2.236)	0.589
	t (95% CI)	−0.795 (−14.670–6.435)	−0.639 (−16.004–8.357)		
Treatment satisfaction					
VAS-satisfaction	PNE	-	8.34 ± 1.33	-	-
	PTRP	-	7.87 ± 1.63	-	-
	t (95% CI)	-	−0.922 (−1.511–0.569)	-	-

^(a)^ Paired t-test. ^(b)^ Cohen’s d. ^(c)^ Independent *t*-test. PNE, pain neuroscience education; PTRP, physical therapy rehabilitation protocol; VAS, visual analog scale. * *p* < 0.05, ** *p* < 0.01, *** *p* < 0.001, statistically significant difference.

## Data Availability

Not applicable.
